# AeQTL: eQTL analysis using region-based aggregation of rare genomic variants

**Published:** 2021

**Authors:** Guanlan Dong, Michael C. Wendl, Bin Zhang, Li Ding, Kuan-lin Huang

**Affiliations:** 1 Department of Biomedical Informatics, Harvard Medical School, Boston, MA 02115, USA; 2 Department of Medicine, McDonnel Genome Institute, Washington University in St. Louis, St. Louis, MO 63108, USA; 3 Department of Genetics and Genomic Sciences, Center for Transformative Disease Modeling, Icahn School of Medicine at Mount Sinai, New York, NY 10029, USA

**Keywords:** Gene expression, Sequencing, eQTL, Rare variants, Data integration

## Abstract

Concurrently available genomic and transcriptomic data from large cohorts provide opportunities to discover expression quantitative trait loci (eQTLs)—genetic variants associated with gene expression changes. However, the statistical power of detecting rare variant eQTLs is often limited and most existing eQTL tools are not compatible with sequence variant file formats. We have developed AeQTL (Aggregated eQTL), a software tool that performs eQTL analysis on variants aggregated according to user-specified regions and is designed to accommodate standard genomic files. AeQTL consistently yielded similar or higher powers for identifying rare variant eQTLs than single-variant tests. Using AeQTL, we discovered that aggregated rare germline truncations in *cis* exomic regions are significantly associated with the expression of *BRCA1* and *SLC25A39* in breast tumors. In a somatic mutation pan-cancer analysis, aggregated mutations of those predicted to be missense versus truncations were differentially associated with gene expressions of cancer drivers, and somatic truncation eQTLs were further identified as a new multi-omic classifier of oncogenes versus tumor-suppressor genes. AeQTL is easy to use and customize, allowing a broad application for discovering rare variants, including coding and noncoding variants, associated with gene expression. AeQTL is implemented in Python and the source code is freely available at https://github.com/Huang-lab/AeQTL under the MIT license.

## 1. Introduction

Advances in sequencing technologies have enabled the generation of large-scale disease cohorts with concurrently available genomic and transcriptomic data^1,2^. Samples with concurrent DNA- and RNA-sequencing (DNA-seq and RNA-seq) provide opportunities to discover expression quantitative trait loci (eQTLs), i.e. genetic variants associated with variations in gene expression^3^. Most existing eQTL tools focus on applying various statistical models to test for association between individual pairs of a variant and the associated gene expression^4–6^. However, for rare variants, the underlying power of the statistical testing is often limited and identifying eQTLs from rare variants remains a challenge^7^.

Multiple methodologies and tools using aggregation strategies to group and identify rare variants associated with disease status have been developed^8–11^, yet similar strategies have rarely been implemented for identifying eQTLs. In addition, these tools are not readily compatible with standard variant call files resulted from sequencing data, including VCFs/MAFs and RNA-seq data from large cohorts.

Here, we present AeQTL, a software tool that performs eQTL analysis on aggregated variants in specified genomic regions and is designed to accommodate standard file formats generated from sequencing data. Previous studies have found that rare germline variants are significantly enriched at both high and low extremes of gene expression in promoter regions^12^. Here, we show AeQTL’s aggregation algorithm can increase the statistical power in order to discover rare variant eQTLs with a larger size of grouped carriers. Further, we demonstrate AeQTL’s capacity in identifying both germline variants and somatic mutations associated with gene expression changes, which can help prioritize disease susceptibility genes or cancer driver genes. In sum, AeQTL offers a much-needed versatile multi-omics tool to integrate DNA-seq and RNA-seq data.

## 2. Methods

**Fig. 1.**
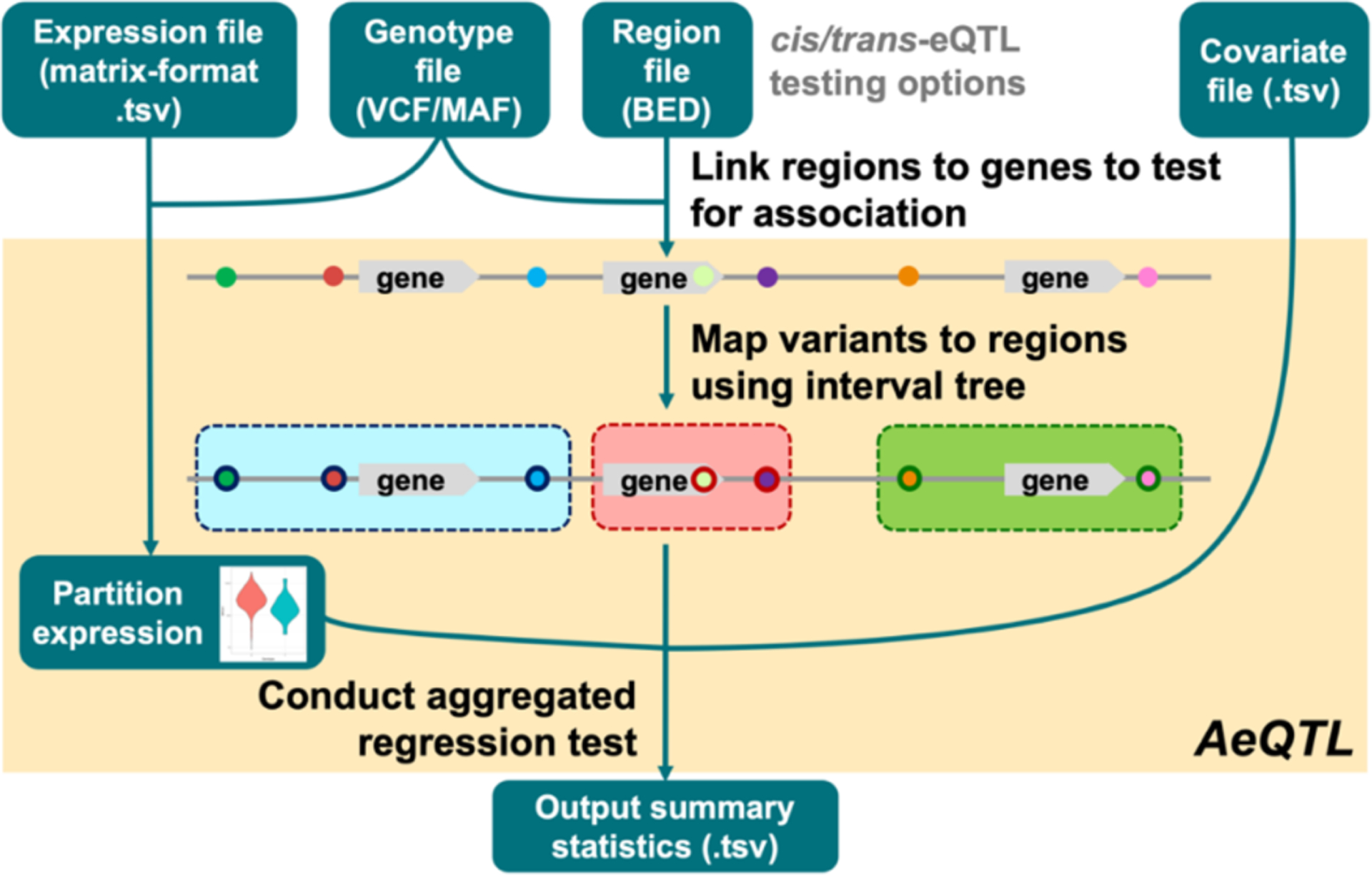
AeQTL workflow. AeQTL links regions to gene expressions to set up the *cis*/*trans-*eQTL testing, partitions sample expression profiles based on aggregated variants in each region, conducts a linear regression test for each region-gene expression pair with optional covariates, and outputs a summary file.

AeQTL implements standard eQTL analysis with user-defined variant-aggregation and its workflow is shown in Fig. 1. AeQTL requires three input files: an expression file, a genotype file, and a region file. The user can provide an additional covariate file for advanced analyses.

### 2.1. Set up eQTL association tests

The input region file (i.e. a BED file) is provided by the user to set up desired association tests between gene expressions and variants. Each line of the file contains a genomic region followed by one or more genes to be tested against. An association test will be set up between the expression level of each specified gene and aggregated variants in the genomic region. If no genes are specified, AeQTL by default will test each region against every gene’s expression in the expression file in a *trans*-eQTL discovery mode. This user-constructed BED file allows flexibility in the design of eQTL analysis for testing both *cis-* and *trans-* eQTLs. We also provide a coding exomic region BED file on our Github page, which can be used for testing and exploratory purposes.

While all variants with matching samples in the expression file will be included in the tests, users can further restrict the aggregation by setting two optional thresholds: the number of mutated samples per region and the number of variants per region, in which case regions with samples or variants below the thresholds will be filtered out. Both thresholds are set to 1 by default.

### 2.2. Aggregate variants and conduct regression analysis

AeQTL aggregates variants by finding overlaps between variants and regions using the interval tree data structure, which is part of the bx-python package (https://github.com/bxlab/bx-python). We used a standalone wrapper of the interval tree (https://github.com/ccwang002/bx_interval_tree) for easier compilation. The interval tree is designed for fast intersect queries on one-dimensional intervals. Compared to other simple positional intersection methods, the interval tree has two major strengths: (1) it allows each interval to be annotated and the annotations will be preserved in queries; (2) the interval tree is implemented in Cython which is faster and more computationally efficient. AeQTL creates an interval tree for each chromosome. For each genomic region provided in the BED file, an interval is specified by the start and end positions, annotated by the region name, and added to the interval tree of its corresponding chromosome. Then, AeQTL finds the intervals that overlap with the given variant to extract its region name and aggregates variants of the same region. AeQTL accommodates different types of variants including single-nucleotide variants (SNVs), insertions, and deletions. After aggregation, AeQTL maps each region to samples and defines a regional mutation status by assigning a genotype “1” if a sample has any variants in this region and a genotype “0” otherwise.

For each tested gene in each region, AeQTL performs a linear regression analysis of RNA-seq gene expression *e* against regional genotype *g*:

$$e=\alpha +\beta g+\epsilon ,\;\epsilon \sim i.i.d.\;\;N\left(0,\;\sigma^{2}\right).$$

The linear model is built using the ordinary least squares method with a residual term *ϵ* that follows a normal distribution with a mean of zero and a constant variance. AeQTL supports covariates *c* to be incorporated into the regression model:

$$e=\alpha +\beta_{1}g+\beta_{2}c+\epsilon ,$$

which enables the model to account for clinical factors and population structures.

### 2.3. Output intermediate mapped files and a result file with summary statistics

AeQTL outputs mapped files with variant genotypes, expression, and covariates for each region, which can be readily routed into other aggregational statistical tests such as SKAT^8^ to allow comparison. Notably, most of the other aggregational software do not allow common sequence file formats (i.e. VCF or MAF) and thus the intermediate files enable flexibility for users.

All the regression results are compiled in a summary file where AeQTL reports both *p*-values and coefficients of the intercept and all dependent variables, including regional genotype and covariates. To correct for multiple testing, AeQTL also reports adjusted *p*-values with false discovery rate (FDR) based on the Benjamini-Hochberg (BH) procedure.

## 3. Results

### 3.1. AeQTL algorithm development and power simulation

**Fig. 2.**
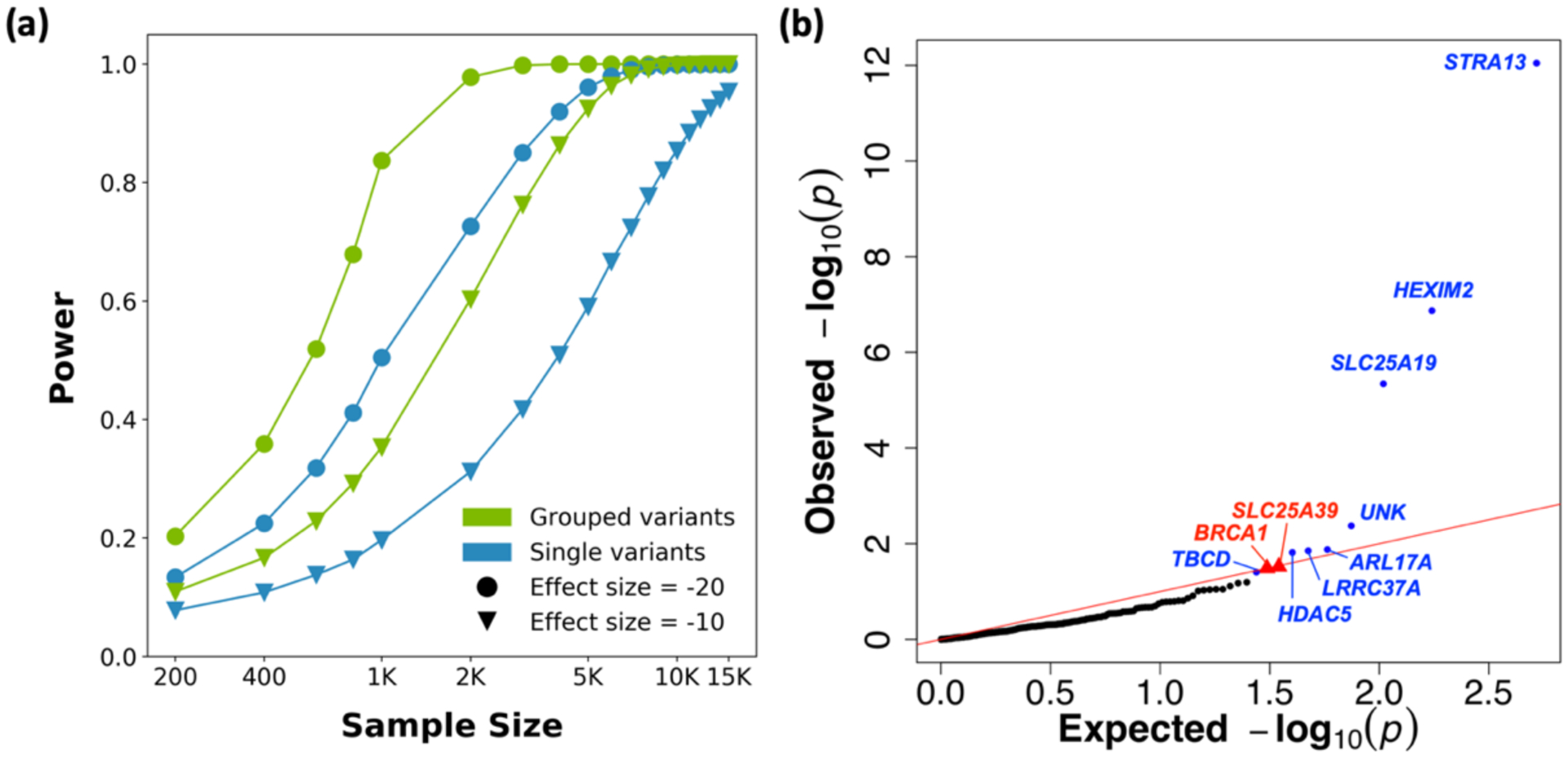
(a) Power simulation on eQTL analyses using rare variants. The statistical powers of AeQTL (grouped variants; in green) and single-variant testing (in blue) are compared under different sample sizes. (b) QQ-plot of −log10 adjusted genotype *p*-values from rare germline truncations on chromosome 17 in breast cancer patients. The red diagonal line is the expected value. *BRCA1* and *SLC25A39* are marked in red triangles. Other genes showing significant associations (P < 0.05) are also labeled and marked in blue.

To demonstrate the aggregating effect on the statistical power of identifying rare variant eQTLs, we performed a simulation analysis using AeQTL. Based on VCF files and expression matrices of 10 rare variants (frequency = 0.1%, five were effective) with a series of sample sizes, we ran AeQTL on both single variants and grouped variants specified by BED files (Fig. 2a). Gene expression profiles were generated from a normal distribution with a mean of 20 and a standard deviation of 10, while effective variants had an effect size t (t = −10 or t = −20) from a normal distribution with a mean of t and a standard deviation of | t ⁄ 2 |. For each sample size, power was calculated as the averaged value of 10,000 independent simulations.

Overall, statistical analysis of aggregated variants consistently demonstrated comparable or higher powers than individual variants. When the sample size was small, the powers of grouped and single variants were similarly low for both effect sizes. As the sample size increased, the powers under all testing conditions increased as expected. However, the powers of grouped variants increased noticeably faster than those of single variants. When effect size = −20, the increased power fold change provided by the AeQTL aggregation method was the most substantial within the sample size interval of 600 to 2,000. The power of grouped variants reached 97% with sample size = 2,000, while single variants required three times the sample size to reach a similar power. When effect size = −10, the power of grouped variants reached 95% with sample size = 6,000 and single variants did not reach the same power until sample size = 15,000. At a sample size of 5,000, the powers of all testing conditions except for single variants with effect size = −10 were higher than 90% and were saturated (> 99%) when the sample size reached 8,000.

### 3.2. Germline eQTL detection

**Fig. S1.**
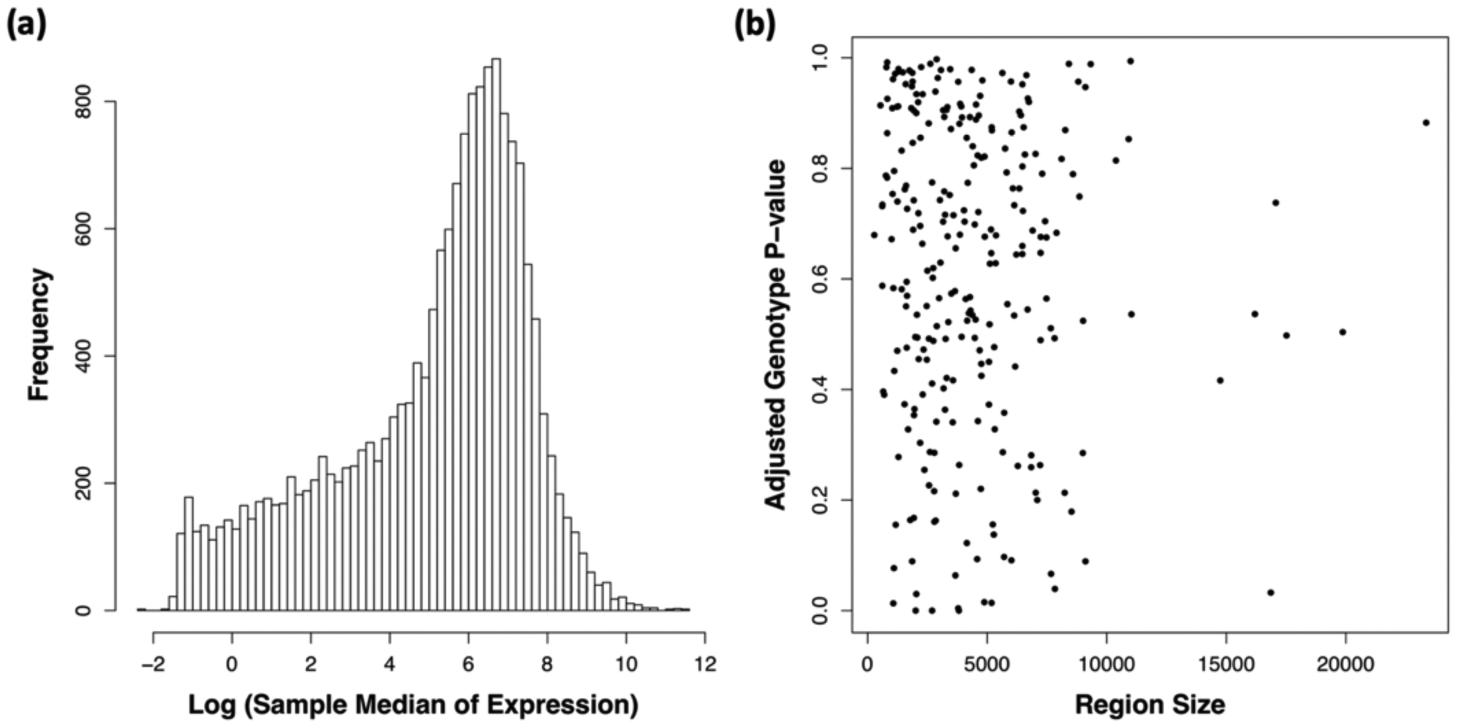
(a) Histogram of the log-transformed sample median gene expression. (b) Sensitivity analysis of whether genomic region size affects eQTL detection. A randomly scattered pattern is shown when adjusted genotype *p*-values are plotted against region sizes. The Spearman correlation test also shows no significant correlation (r_s_ = 0.16, P = 0.18).

We further tested AeQTL on rare germline truncations (minor allele frequency ≤ 0.05%) on chromosome 17 of the TCGA PanCanAtlas cohort^13^. We tested the hypothesis that rare truncations in cancer susceptibility genes are associated with their *cis*-expression in tumor samples. For the input BED file, we specified each of the genes on chromosome 17 as a region of interest and tested truncations in each gene region against the expression of its located gene. We used the level 3 TCGA RNA-seq gene expression data in RSEM^14^ from breast invasive carcinoma (BRCA) patients and incorporated six covariates: age, gender, ethnicity, tumor stage, as well as the top two components from the principal component (PC) analysis on population structure (accounting for > 80% of the top 20 PCs). Because low gene expression levels would likely present technical noises, we filtered out genes with median expressions lower than log(2) (Fig. S1a). This germline analysis, which contained 1,071 samples, 3,150 variants, and 261 unique gene regions, took ~35 min on a Mac with a 2.3 GHz processor and 8 GB memory.

We visualized the distribution of adjusted genotype *p*-values on a QQ-plot (Fig. 2b). The expression of *BRCA1* was significantly associated with aggregated rare truncations in the *BRCA1* exomic region (P = 0.033) in the BRCA cohort, demonstrating that AeQTL could efficiently identify grouped genotype-expression association. In addition, *SLC25A39* (P = 0.030) was among the top-ranked genes whose expressions were negatively associated with the aggregated rare truncations in their regions. We also carried out a sensitivity analysis of adjusted genotype *p*-values against region sizes, which suggested no significant correlation between the two, indicating the lack of false-discovery from large genes (r_s_ = 0.16, P = 0.18, Fig. S1b).

### 3.3. Somatic eQTL detection

Aside from germline variants, we also tested AeQTL on somatic truncations and missense mutations across 32 cancer types of the TCGA PanCancer cohort^15^. Similar to the germline eQTL detection, the input BED file contained coding sequence positions of all genes where each gene region was tested against the expression of itself in a *cis*-expression pattern. We used the TCGA PanCancer RNA-seq data and incorporated seven covariates: age, gender, ethnicity, the same top two PCs on population structure as in the germline analysis, cancer subtype, and whether the patient showed an onset age ≤ 50 years old. A separate AeQTL run was performed for each variant type in each cancer type, and all the output summary files for each variant type were compiled together for multiple testing correction using FDR to generate the final pan-cancer output.

**Fig. 3.**
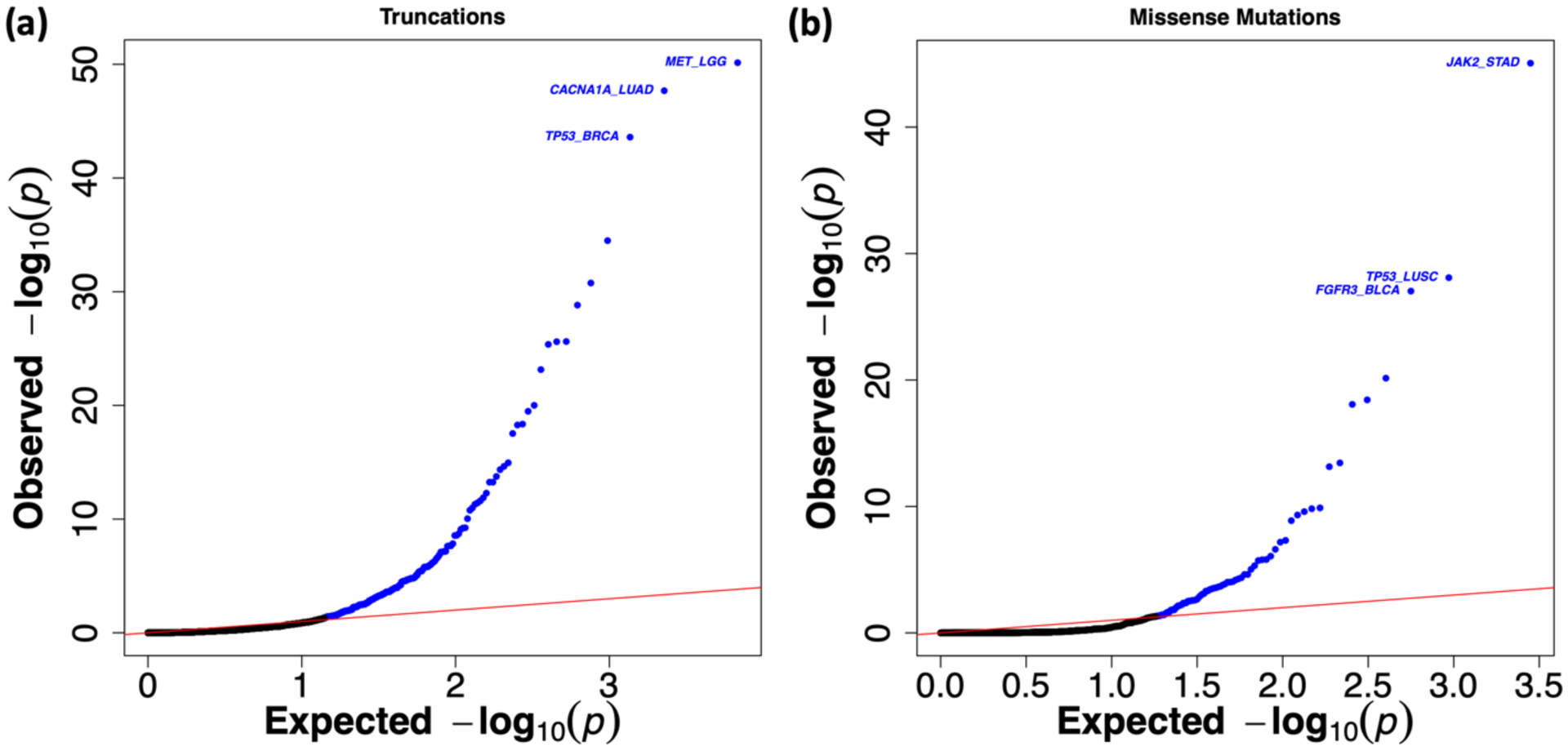
QQ-plots of −log10 adjusted genotype *p*-values from somatic truncations (a) and missense mutations (b) on likely driver genes in 32 cancer types. The red diagonal line is the expected value. Gene-cancer pairs showing significant associations (P < 0.05) are marked in blue and the top three ranked pairs are labeled.

We interrogated a subset of 299 genes that were reported as likely driver genes by Bailey et al.^16^ and extracted 23,849 truncations and 11,966 missense mutations located in these genes from 8,639 samples. AeQTL identified 243 gene-cancer pairs with truncations and 77 gene-cancer pairs with missense mutations that were significantly associated with their respective gene expressions (FDR < 0.05, Fig. 3). The total and unique variant sites used in the analysis are summarized in Table S1. The top-ranked gene-cancer pairs with truncations include the *MET* proto-oncogene from brain lower grade glioma (LGG), the calcium channel gene *CACNA1A* from lung adenocarcinoma (LUAD), and *TP53* from BRCA; the top-ranked gene-cancer pairs with missense mutations include *JAK2* from stomach adenocarcinoma (STAD), *TP53* from lung squamous cell carcinoma (LUSC), and *FGFR3* from bladder urothelial carcinoma (BLCA).

To demonstrate the computational capacity of AeQTL, we expanded the analysis to the entire dataset, including 335,866 truncations and ~2 million missense mutations from 10,208 samples. AeQTL identified 1,179 gene-cancer pairs with truncations and 3,241 gene-cancer pairs with missense mutations significantly associated with their respective gene expressions (FDR < 0.05).

For significant gene-cancer pairs with truncations, 156 overlapped with the likely driver genes. For significant gene-cancer pairs with missense mutations, 115 overlapped with the likely driver genes. Interestingly, we also identified many top-ranked genes that were not previously identified drivers by TCGA PanCanAtlas driver project^16^. The top-ranked somatic eQTL genes with truncations include *OR8D1* in LUSC, *SOX10* in head and neck squamous cell carcinoma (HNSC), and *PSG7* in kidney renal clear cell carcinoma (KIRC). The top-ranked somatic eQTL genes with missense mutations include *USP29* in cholangiocarcinoma (CHOL) and *AMELX*, *CNTN5*, and *OR1L3* in lymphoid neoplasm diffuse large B-cell lymphoma (DLBC). Multiple recent reports highlight the functionality of the “long-tail driver genes” found with lesser mutations in multiple cancer types^17–21^. These somatic eQTL genes and their expression-associated mutations represent new candidates that warrant further investigations.

### 3.4. eQTL patterns of oncogenes and tumor suppressor genes

Cancer driver genes, depending on their mutated cancer type and pathway context, can be subclassified into oncogenes and tumor suppressor genes (TSGs). But most existing methods to classify oncogenes and TSGs leveraged cohort-level mutation data^22–25^ that lack considerations of their downstream consequences. To understand whether eQTL patterns could capture the distinction between oncogenes and TSGs, we further investigated the significant genes classified as oncogene or TSG from Bailey et al.’s DNA mutation-based study^16^.

**Fig. 4.**
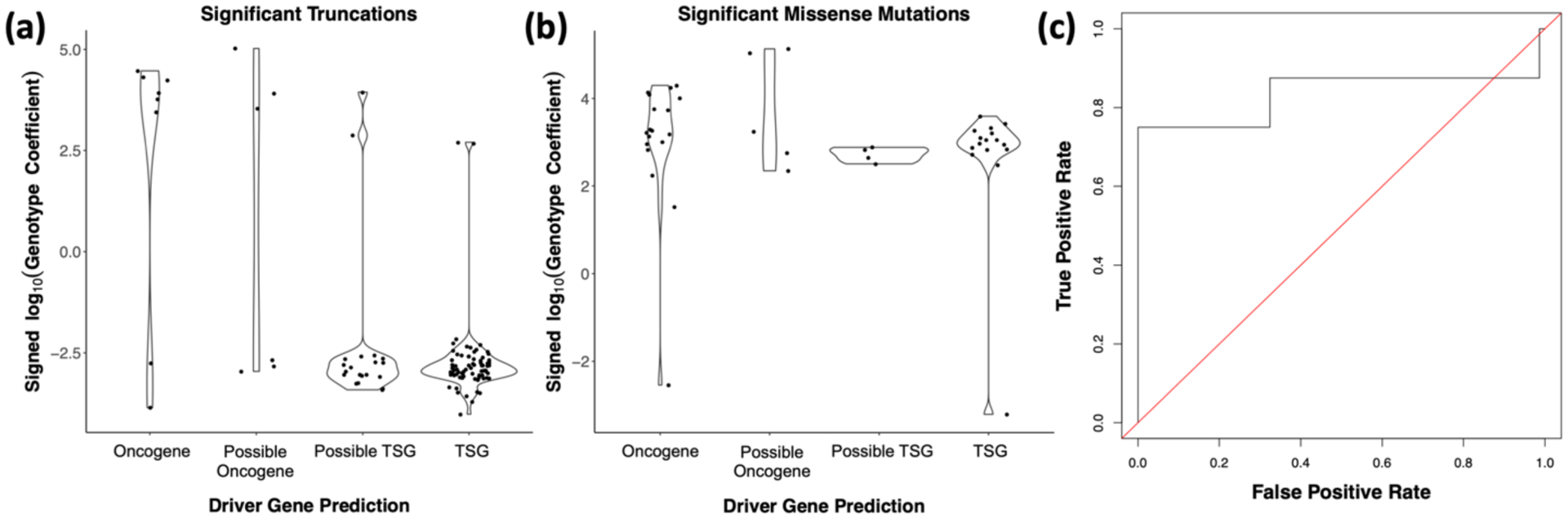
Violin plots of signed log10 genotype coefficients from significant somatic truncations (a) and missense mutations (b) on likely driver genes in 32 cancer types (P < 0.05). The driver gene predictions are obtained from Bailey et al. and genes with no predictions are filtered out. (c) ROC curve of significant somatic truncations labeled as either “Oncogene” or “TSG” (AUC = 0.863). Genes labeled as “Possible Oncogene” or “Possible TSG” are filtered out.

In the likely-driver-gene subset analysis, the genotype coefficients of truncations showed a strong association with their respective predicted classifications of oncogenes or TSGs. Genes predicted to be oncogenes or possible oncogenes had larger positive genotype coefficients while genes predicted to be TSGs or possible TSGs had larger negative genotype coefficients (Fig. 4a), demonstrating a polarized pattern of how truncations in oncogenes versus TSGs may affect their respective genes’ expression in opposite directions. Moreover, we performed a receiver operating characteristic (ROC) analysis evaluating how well genotype coefficients could predict the labels of driver genes. The analyses yielded an area under the curve (AUC) of 86.3% (Fig. 4c), suggesting the potential of using somatic truncations eQTL patterns to distinguish between oncogenes and TSGs. In comparison, such a pattern was not recapitulated in the genotype coefficients of missense mutations, where both oncogene and TSG mutations were associated with increased gene expressions (Fig. 4b). Overall, genotype-expression analyses revealed distinct eQTL patterns associated with missenses versus truncations and oncogenes versus TSGs in cancer drivers.

### 3.5. Comparison with existing variant-aggregation methods

**Fig. S2.**
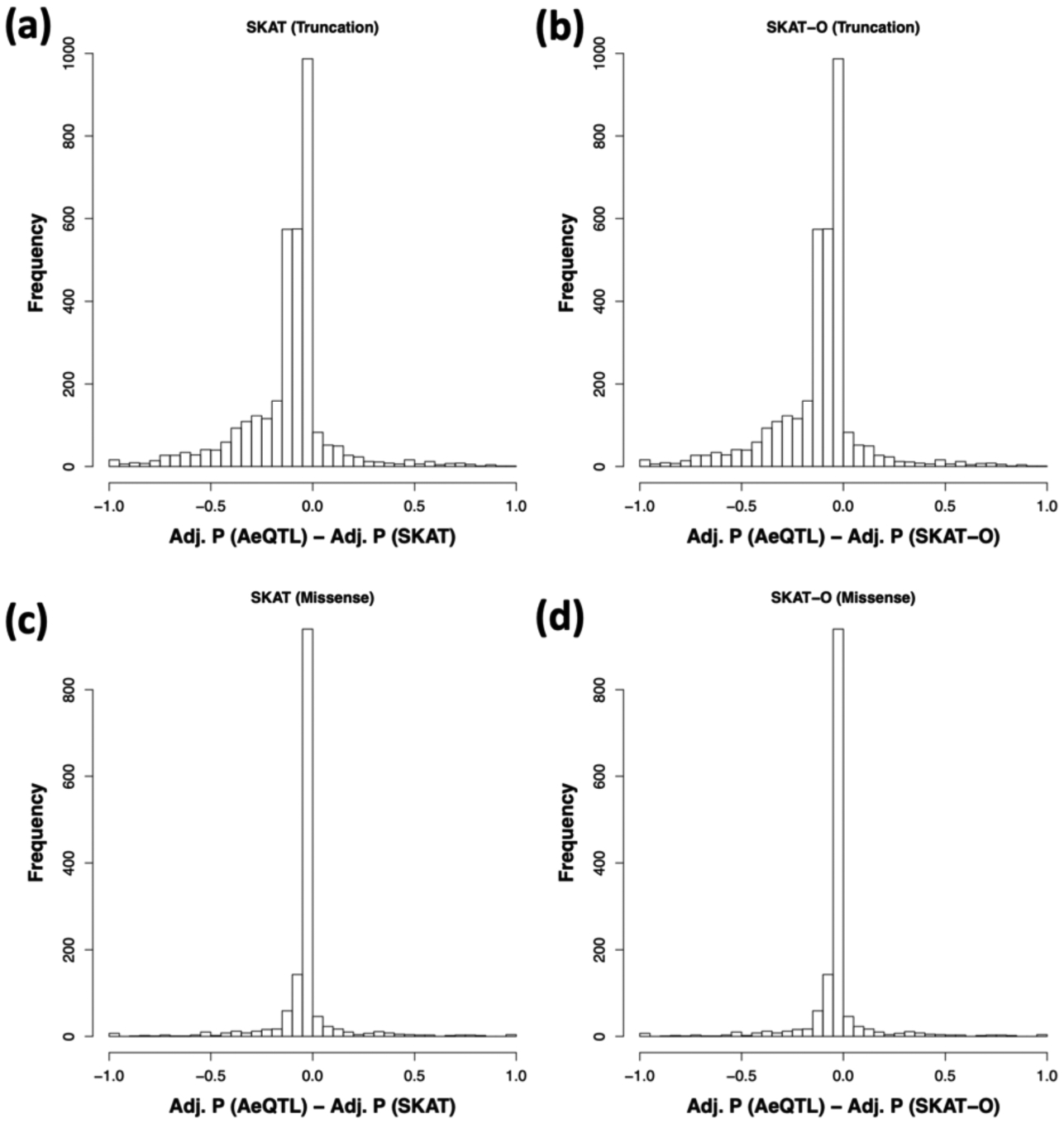
Histograms of the differences between adjusted *p*-values from AeQTL and (a) SKAT for truncations, (b) SKAT-O for truncations, (c) SKAT for missense mutations, (d) SKAT-O for missense mutations in TCGA somatic likely-driver-subset analysis.

**Fig. S3.**
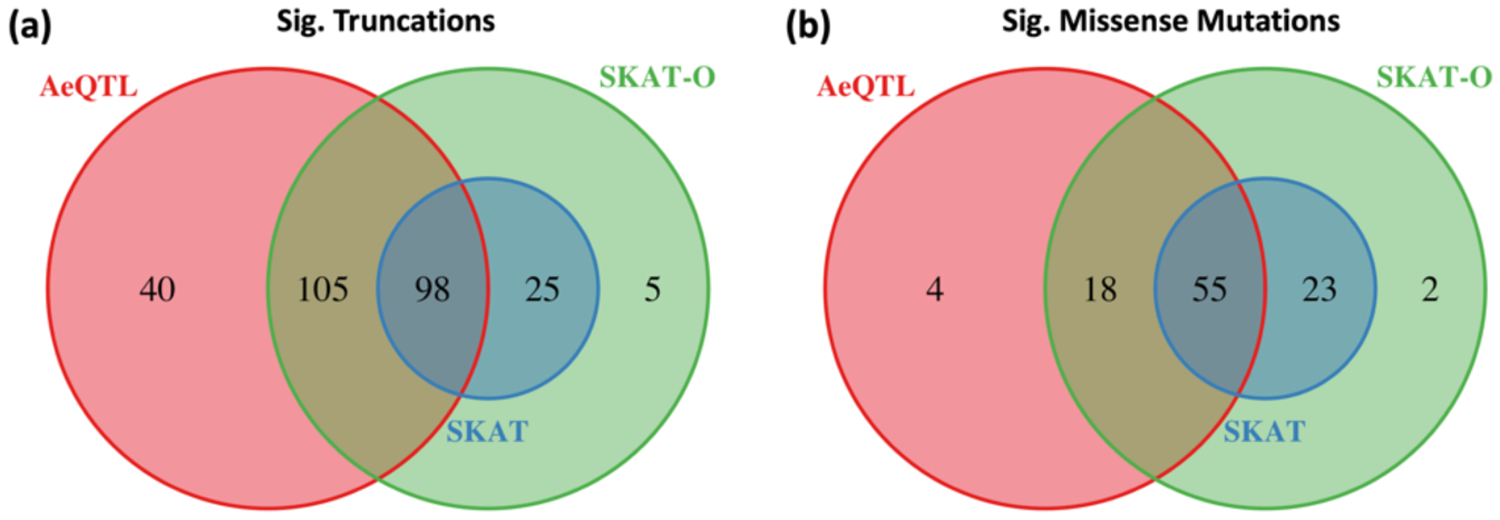
Venn diagrams of significant associations identified by AeQTL, SKAT, and SKAT-O for (a) truncations and (b) missense mutations in TCGA somatic likely-driver-subset analysis.

We used the intermediate mapped files from TCGA somatic likely driver subset to test two of the most popular variant-aggregation methods, SKAT^8^ and SKAT-O^9^, and performed the same multiple testing correction based on the BH procedure using FDR. For each gene-cancer pair, we analyzed the difference between the adjusted p-value from AeQTL and the adjusted p-values from SKAT and SKAT-O. The majority of the adjusted p-values from AeQTL were lower than the ones from SKAT and SKAT-O (median difference for truncations = −0.076 (SKAT) and −0.017 (SKAT-O), median difference for missense = −0.012 (SKAT) and −0.010 (SKAT-O), Fig. S2). For both truncations and missenses, SKAT-O identified more significant associations than SKAT while recapturing the ones identified by SKAT. This is not surprising since SKAT-O leverages both SKAT and burden test and implements a small-sample adjustment procedure, which should work well with somatic data. Notably, AeQTL was able to identify more significant truncation associations than SKAT-O and 40 out of the 243 associations were unique to AeQTL (Fig. S3a). On the other hand, SKAT-O identified more significant missense associations than AeQTL (Fig. S3b). This is possibly due to SKAT-O’s better compatibility with scenarios where only a fraction of variants show functionalities and potentially different directionalities. Further, neither SKAT nor SKAT-O provides a regression coefficient for regional genotype, which makes it difficult to understand the direction of variant’s effect on gene expression and make discoveries such as the polarized eQTL patterns of oncogenes and TSGs.

Most existing variant-aggregation methods are designed to conduct association tests on quantitative traits, most notably for SNP-array genotype data. While each gene expression value can be considered as a continuous trait for analyses using these methods, few of those readily accommodate sequencing data formats such as large VCFs/MAFs and expression matrices from cohorts. To address this challenge, AeQTL can complement the existing methods since it provides intermediate mapped files which can be routed into other aggregational statistical tests based on the users’ preference and hypothesis. We believe such user-friendly functionality would be essential to help the field adopt aggregated eQTL testing from sequencing data.

## 4. Discussion

AeQTL increases the power of eQTL detection by aggregating variants in a defined genomic region. We have applied AeQTL to both synthetic and real datasets. The synthetic dataset demonstrated that variant aggregation consistently yielded similar or higher powers for rare variant eQTL detection. For real datasets, we used rare germline truncations in breast cancer to showcase that AeQTL can efficiently identify significant associations between grouped variants and gene expressions. Furthermore, we applied AeQTL to somatic mutations in a pan-cancer dataset and identified top-ranked gene-cancer pairs that were significantly associated with either truncations or missense mutations in their respective gene regions.

To facilitate users’ adoption of AeQTL, we also provide input files to conduct analyses using MAF datasets, as used by TCGA PanCancer somatic mutation data^15^. The application procedure is described in detail and included as an example on Github.

AeQTL is easy to use and customize. Out of the three required input files (region, variant, and expression files), both variant and expression files can be directly taken by AeQTL without any complicated reformatting or pre-processing, while the user-constructed region file allows great flexibility for setting up association tests. Moreover, we provide the exome BED file used in our TCGA analyses on the Github page so that users can easily explore the tool in the *cis*-eQTL mode.

The simplicity of AeQTL’s method design means that it can be broadly applied to datasets without imposing on them excessive assumptions or limitations. We have demonstrated AeQTL’s promising performance when applied to cancer datasets. However, with more genomic and transcriptomic data being collected and made available in other fields such as neurodegenerative diseases and psychiatric diseases, we believe AeQTL will contribute to multiple areas of study. Aside from research, another important application of AeQTL is in educational settings. From processing standard sequencing data formats, to building classic regression models, and to producing FDR-controlled outputs, AeQTL has a clear and simple workflow that can facilitate the learning process of eQTL analysis.

There are a few aspects of the method that may be improved. First, a potential downside of having a simple method that suits more datasets is that the aggregated genotype of each region is not weighted. Having unweighted variants does not necessarily lead to worse performance, since the underlying mechanism is often unknown and having preset weights may actually confound the results. Nevertheless, we would like to offer more options for users in cases where there are known variations in the magnitudes of effect for certain variants. We plan to introduce more optional settings such as an annotated variant file with a scaling factor, either specified by the user or generated using other algorithms.

Traditional methods to classify oncogenes or tumor suppressors rely on algorithms considering only DNA-mutation patterns or functional curation^22–25^. Herein, we present truncation eQTL patterns revealed by AeQTL as a potential new method to distinguish oncogenes (elevated expression) from tumor suppressors (reduced expression). In TSGs, truncations including nonsense variants or frameshift variants may introduce early stop-codons that likely have led to nonsense-mediated decay (NMD), thus abolishing gene transcripts. In contrast, oncogene truncations show a higher frequency of inframe indels^16^, albeit the mechanisms through which they are associated with higher gene expression warrant further investigation.

With increasingly available cohorts of matched genomic (e.g. whole-genome sequencing) and transcriptomic (e.g. RNA-seq) data, we expect that the robust and versatile AeQTL tool can be applied broadly for discovering rare coding and noncoding variants associated with gene expression.

## 6. Appendix

**Table S1.** Summary of the number of variant sites used in TCGA somatic likely-driver-subset analysis.

	Likely Driver Genes	Likely Driver Genes (sig.)
**Unique Truncations**	15430	4190
**Total Truncations**	18124	5311
**Unique Missense Mutations**	5233	1364
**Total Missense Mutations**	9882	3059
